# Multilevel Analysis of Trachomatous Trichiasis and Corneal Opacity in Nigeria: The Role of Environmental and Climatic Risk Factors on the Distribution of Disease

**DOI:** 10.1371/journal.pntd.0003826

**Published:** 2015-07-29

**Authors:** Jennifer L. Smith, Selvaraj Sivasubramaniam, Mansur M. Rabiu, Fatima Kyari, Anthony W. Solomon, Clare Gilbert

**Affiliations:** 1 Department of Disease Control, Faculty of Infectious and Tropical Diseases, London School of Hygiene & Tropical Medicine, London, United Kingdom; 2 Global Health Group, University of California San Francisco, San Francisco, California, United States of America; 3 Medical Statistics Team, Division of Applied Health Sciences, University of Aberdeen, United Kingdom; 4 Prevention of Blindness Union, Riyadh, Saudi Arabia; 5 Department of Clinical Research, Faculty of Infectious and Tropical Diseases, London School of Hygiene & Tropical Medicine, London, United Kingdom; 6 College of Health Sciences of University of Abuja, Abuja, Nigeria; University of California San Francisco, UNITED STATES

## Abstract

The distribution of trachoma in Nigeria is spatially heterogeneous, with large-scale trends observed across the country and more local variation within areas. Relative contributions of individual and cluster-level risk factors to the geographic distribution of disease remain largely unknown. The primary aim of this analysis is to assess the relationship between climatic factors and trachomatous trichiasis (TT) and/or corneal opacity (CO) due to trachoma in Nigeria, while accounting for the effects of individual risk factors and spatial correlation. In addition, we explore the relative importance of variation in the risk of trichiasis and/or corneal opacity (TT/CO) at different levels. Data from the 2007 National Blindness and Visual Impairment Survey were used for this analysis, which included a nationally representative sample of adults aged 40 years and above. Complete data were available from 304 clusters selected using a multi-stage stratified cluster-random sampling strategy. All participants (13,543 individuals) were interviewed and examined by an ophthalmologist for the presence or absence of TT and CO. In addition to field-collected data, remotely sensed climatic data were extracted for each cluster and used to fit Bayesian hierarchical logistic models to disease outcome. The risk of TT/CO was associated with factors at both the individual and cluster levels, with approximately 14% of the total variation attributed to the cluster level. Beyond established individual risk factors (age, gender and occupation), there was strong evidence that environmental/climatic factors at the cluster-level (lower precipitation, higher land surface temperature, higher mean annual temperature and rural classification) were also associated with a greater risk of TT/CO. This study establishes the importance of large-scale risk factors in the geographical distribution of TT/CO in Nigeria, supporting anecdotal evidence that environmental conditions are associated with increased risk in this context and highlighting their potential use in improving estimates of disease burden at large scales.

## Author Summary

Trichiasis (TT) and corneal opacity (CO) are chronic stages of trachoma, which remains an important cause of blindness. This study used multilevel spatial models to investigate risk factors for TT/CO in Nigeria, including data for more than 13,500 adults aged 40 years and above collected in the 2007 National Blindness and Visual Impairment survey. Individual-level risk factors were consistent with those identified in other studies, including a higher risk in females, older individuals and those with lower socioeconomic status. After controlling for these factors, there was evidence that a number of environmental and climatic factors are associated with the distribution of TT/CO in Nigeria. These findings establish for the Nigerian context the importance of risk factors at different scales for the later stages of trachoma, supporting anecdotal evidence that hotter, drier environmental conditions are associated with increased risk.

## Introduction

Trachoma is the leading infectious cause of blindness worldwide, most recently estimated to be responsible for the loss of 333,000 disability-adjusted life years (DALYs) in 2010 [1]. Recurrent episodes of infection with the bacterium *Chlamydia trachomatis* and associated inflammation cause cumulative scarring of the under surface of the upper eyelid which, in some individuals, eventually leads to trichiasis–a clinical stage of trachoma where the eyelashes turn inwards and touch the eye. Without surgical intervention, this condition can progressively damage the cornea and lead to visual impairment and irreversible blindness later in life [2,3].

While a number of studies have identified risk factors that are associated with clustering of trachoma within villages, households [4–7] and individuals [8], only a few studies have quantified associations at larger scales [9,10]. Anecdotally, trachoma is believed to be a greater public health risk in dry, dusty and hot environments, although disease may be present anywhere that overcrowding and poor hygiene allow transmission [11,12]. Climatic variables are postulated to indirectly influence the transmission of trachoma through the following mechanisms: low rainfall which leads to reduced access or use of water for washing faces; higher temperatures which may influence the distribution and activity of the vector *Musca sorbens*; and climatic conditions that favour drying of faeces, the fly’s preferred breeding site [13–17]. In addition, there may be a potential role for ocular dryness or environmental irritants to contribute to progression of chronic disease, by aggravating scarring processes [2,18–20].

However, robust studies investigating relationships between detailed epidemiological observations and environmental determinants are scarce. Existing studies provide some support for a role of temperature and rainfall in the distribution of trachoma [9,21–23], as well as altitude (which might be a proxy for temperature) [24–26]. However, most studies are limited by lack of control for individual level factors [21,25,27], and in particular variation in socioeconomic factors [28]. In practice, it is difficult to disentangle the effects of risk factors of trachoma at different spatial levels, due to a complex interplay between large-scale factors such as climate, and mediating factors at smaller scales, like water availability, sanitation access at the household level and individual behaviours, including household water use and personal hygiene [26,29,30].

Bayesian hierarchical models (BHM) are a robust and well established methodology for modelling data that are naturally grouped, and for identifying risk factors at different scales [31]. This approach can be expanded to incorporate information on residual underlying spatial patterns, thus explicitly addressing any remaining spatial correlation between observations that may affect estimates of standard errors [32]. Previous studies have used this approach to identify risk factors at multiple hierarchical levels for a variety of tropical diseases, using data from school-based and community surveys, including malaria [33], soil-transmitted helminths[34,35], schistosomiasis [36,37] and trachoma [9]. A common application in multilevel models is to then apportion the variance in the response according to the different levels of the data, referred to as the variance partition coefficient (VPC) [38,39]. These methods offer a robust and flexible approach to modelling prevalence data routinely collected as part of disease control programmes in developing countries.

Nigeria is a populous country with over 160 million people, comprising approximately 20% of the total population in Africa [40]. There are diverse climatic conditions across the country, and three broad ecological zones: the southern rainforest zone, the central Guinea Savannah zone and the semi-arid northern Sudan Savannah [41]. Trachoma is a significant public health problem in the north of the country and currently only 43% of districts suspected to be endemic have been surveyed by population based prevalence surveys [42]. The 2007 National Blindness and Visual Impairment Survey was conducted in Nigeria to provide estimates of the prevalence and causes of blindness at the national level in order to inform policy and planning for the elimination of avoidable blindness [43]. During this survey, participants were assessed for the presence of trachomatous trichiasis (TT) and corneal opacity attributed to trachoma (CO), providing a unique opportunity to describe the distribution of later stages of trachoma in relation to underlying risk factors in Nigerian adults.

This analysis aims to use geostatistical BHMs to quantify the relationship between climatic factors and trachomatous trichiasis or corneal opacity (TT/CO) amongst adults in Nigeria, while accounting for the effects of risk factors at other levels and any residual spatial correlation.

## Methods

### Overview

Available data included field collected data at the individual level and remotely sensed or interpolated environmental variables at the cluster level. An exploratory principal components analysis was conducted on all climatic variables with a correlation coefficient ≥0.70, in order to explore covariance and variance between factors and ultimately inform dataset reduction and model building.

All field collected data were used with a reduced set of environmental covariates to build hierarchical multivariate regression models for the presence or absence of TT or CO (Table 1). Model-building took a spatially explicit approach and incorporated geostatistical random effects to account for spatially-structured residual clustering.

**Table 1:** Summary statistics of the reduced set of climatic and environmental covariates included in model building.

Variable	Median (range)^a^	SD
**Climate**		
Land surface temperature (LST) (°C)	31.7 (22.5, 42.8)	5.0
Mean annual temperature (°C)	26.4 (21.8, 28.7)	1.0
Mean annual precipitation (mm)	1284.0 (407.0, 3833)	639.2
**Environmental**		
Altitude (meters)	270.5 (4.0, 1287.0)	226.1
Savannah or Grasslands^b^	17.0%	-
Ruminant density (animals per 5km cell)	68.1 (0, 1051.4)	144.8
Cost-distance to road network	1387.5 (0, 22842.7)	2983.3
Urban classification	27.3% urban	-
Population density	285.0 (8.0, 27982.0)	2570.4
Enhanced vegetation index (EVI)^c^	0.3 (0.1, 0.5)	0.1

SE: standard deviation; °C: degrees Celsius; mm: millimetres; km: kilometer

^a^ Proportion of sites for binary variables (Savannah/Grasslands and Urban classification);

^b^ Reclassified from global land cover

### Data

#### National blindness survey

Data were collected over a 30-month period from January 2005 to July 2007. The framework of this study has been fully described elsewhere [44]. Briefly, a multistage stratified cluster random sampling strategy with probability proportional to size was employed to generate a nationally representative sample of adults aged 40 years and above. A total of 50 adults were enumerated in each of 305 clusters, using a random walk procedure from the centre. Visual acuity (VA) was assessed and all participants had a basic eye examination by a qualified Nigerian ophthalmologist. The presence or absence of TT and CO were recorded based on diagnoses using the WHO simplified grading scheme [45]. In addition, a questionnaire was administered to collect demographic and socioeconomic indicators for each participant.

One cluster could not be geolocated and was excluded from the analysis. The majority (80%) of clusters had a specific longitude and latitude recorded by GPS during the survey. The remaining 20% of the clusters were geolocated to a specific location using a variety of electronic gazetters (13%) or the centroid of the corresponding local government area (LGA) (7%).

#### Environmental and climatic data

Environmental variables were selected based on their potential relevance to transmission of ocular *C trachomatis* infection, either through water availability, the physiology and behaviour of *M sorbens*, or progression of disease. Gridded data were obtained from a variety of sources, fully detailed in S1 Table and mapped in S1 Fig. These variables included interpolated or satellite data on annual climate trends (mean annual precipitation, land surface temperature, mean annual temperature, annual aridity index and potential evapo-transpiration (PET)) and extreme or potentially limiting climatic factors (maximum temperature in the warmest month, precipitation of driest month). Long-term averages of these indices were considered appropriate as later stages of trachoma are believed to represent the cumulative effects of repeated episodes of active disease over many years. Other environmental factors included altitude, enhanced vegetation index (EVI, sometimes used as a surrogate for rainfall), urbanisation category, land cover type, population density, distance to nearest waterbody and livestock density. Information on gridded environmental and climatic variables was extracted for each point location in R version 2,10,1.

### Data categorization

#### Field collected data

Age was classified into ten year age bands, based on the nonlinear relationship observed with TT/CO and to minimise the effect of reporting biases. Occupation, literacy, water source and latrine type are all characteristics that capture various dimensions of an individual’s socioeconomic status (SES) [46]. These factors may potentially influence transmission of *C trachomatis* through their relationship with overcrowding, water availability and usage, waste disposal and hygiene behaviours. Occupational category was recoded to distinguish professionals, semi-skilled workers and the unemployed. Literacy was kept as three categories based on self-reported ability to read easily, with difficulty or not at all. Presence of a latrine has been associated with lower density of *M sorbens* and fly-eye contact [7,15,47,48] and latrine type was categorized as flush toilet, pit latrine or bush for this analysis. Water source was recoded as a binary variable in two ways: 1) to reflect an individuals’ access to an improved water source, using definitions provided by the Joint Monitoring Programme for Water Supply and Sanitation [49], and 2) to reflect distance and availability of water by categorising water sources located within the household or yard separately from wells, boreholes, bought and surface water.

#### Environmental data

Global land cover was recoded in this analysis to distinguish savannah and grassland areas, which have previously been associated with a higher risk of trachoma [21,27]. Categorical variables for each environmental variable were generated based on quartiles, except where there was a clear pattern in the risk of TT/CO across the factor values to inform classification. All continuous environmental and climatic data were standardised to improve convergence of the models.

### Modelling approach

#### Environmental variable selection

As noted in similar analyses, multicollinearity between environmental variables commonly presents a challenge in model-building [22]. A principal components analysis (PCA), was used to explore the underlying structure of climatic variables, in terms of variance and covariance, and inform reduction of the dimensionality of the dataset for subsequent model building strategies [50]. Principal components were not used directly in the model, as they are less interpretable. However, collinear pairs of climatic variables from each grouping identified in the PCA were added through sequential regression. This approach involves determining a sequence of importance for explanatory variables and removing common variation in order to create a new variable [51,52]. As the literature provides the strongest evidence base for an association between precipitation and trachoma, this was considered the principal climatic factor in the regression [9,21,53].

#### Model building

Initially a non-spatial, frequentist approach was used to select candidate variables for Bayesian spatial models, using binomial logistic regression models with a cluster-level random effect. Univariate analyses of each field-collected variable and the reduced set of environmental variables were conducted to identify initial covariates associated with TT/CO and bivariate analysis used to explore relationships with potential confounders or correlated variables. Univariate models were fitted with continuous and categorical variables in turn, and the variable with the lowest Akaike information criterion (AIC) retained for the modelling process [54]. If included categorically, a model including the categorical variable was compared to one fitted with a quadratic term in addition to the continuous variable.

Initial covariate selection was done using a forward stepwise procedure for each of the two levels (individual and cluster), in order to develop a multivariate multilevel model keeping variables with a p-value of 0.1 or less. As explained above, precipitation was the first climatic variable to be added into the model containing individual-level covariates. After accounting for the common variation captured by this variable, other collinear climatic variables were regressed against it and residuals included as new variables that are conditional on precipitation. Non-linear associations between environmental covariates and the outcome were explored by adding a squared term and assessing model fit. As the majority (79%) of households had only one (39%) or two (40%) individuals included in this study, resulting model instability led to the exclusion of the household level random effect.

#### Bayesian models

Final equivalent Bayesian models were then developed, incorporating a geostatistical random effect. Models took the form:

$$Y_{ij}\sim Bernoulli(p_{ij})$$

$$logit\left(p_{ij}\right)=\alpha +\sum_{g=1}^{n}\beta_{g}\times x_{ij}+\sum_{h=1}^{n}\beta_{h}\times x_{j}+v_{j}+u_{j}$$

Where *Y*_*ij*_ is the infection status of individual *i* in cluster *j*, *p*_*ij*_ is the probability of a positive response, α is the intercept, $\sum_{g\;=\;1}^{n}\beta_{g}\times x_{ij}$ is a vector of *g* independent variables at the individual level measured in the field multiplied by their coefficient *β*_*g, $\;\sum_{h\;=\;1}^{n}\beta_{h}\times x_{j}$*_ is a vector of *h* independent variables at the cluster level multiplied by their coefficient *β*_*h*_, *ν*_*j*_ is a non-spatial random effect (NSRE) and *u*_*j*_ is a spatial random effect (SRE) at the cluster level. Non-informative priors were specified for the intercept (uniform prior with bounds -∞, ∞) and the coefficients (normal prior with mean = 0 and precision, the inverse of variance = 1×10^-6^). NSREs were incorporated into all models, in order to allow residual variation to have uncorrelated and correlated components. The SRE models any residual correlation that is spatially structured using an isotropic, stationary exponential decay function: *f(d*_*ab*_;*ϕ*) = exp[-(*ϕd*_*ab*_)]where *d*_*ab*_ is the straight-line distance between pairs of points *a* and *b*, and *ϕ* is the rate of decline of spatial correlation. The NSRE had a non-informative priors imposed on its variance (uniform distribution with delimiting values of 0.01 and 100).

Non-spatial model residuals were used to construct semi-variograms, which were visually inspected for spatial dependency and non-stationarity, which was incorporated by inclusion of location-specific geostatistical random effects in the northern and southern states. Non-linearity in the final model was assessed by including a squared term in the regression model. A burn in of 20,000 iterations was allowed, followed by 10,000 iterations where values for monitored variables were stored and thinned by 10. Diagnostic tests for convergence of the monitored variables were undertaken, including visual examination of history and density plots. The runs were also assessed for evidence of autocorrelation. Model performance was assessed by comparing deviance information criteria (DIC).

Residuals were checked for normalcy and a sensitivity analysis was run, excluding sites which were geolocated to the local government area (LGA) centre and might introduce error. All analyses were run from R in WinBUGS 1.4 (MRC Biostatistics Unit, Cambridge and Imperial College London, UK), using the package ‘R2WinBUGS’.

### Variance components

There is no simple way to measure variance partition coefficient (VPC) for discrete response multilevel models, as the variance at the two levels are measured on different scales and dependent on individual level predictor variables. We used a simulation approach implemented in R 2,10,1 to estimate the VPC introduced by Goldstein et al. (2002), which approximates the variance at each level from a large number of simulations based on the variance in the second-level random effect, beta values from the non-spatial model and average values for each coefficient [38].

### Ethics statement

Ethical approval for the Nigerian National Blindness Survey was provided by the London School of Hygiene and Tropical Medicine and the Federal Government of Nigeria. The study adhered to the tenets of the Declaration of Helsinki. Written informed consent was obtained from all participants before they were examined. Eye examination and service facilities (including aphakic spectacles if required) were provided to all individuals, regardless of their consent to participate in the study.

## Results

### Raw data

**Fig 1:**
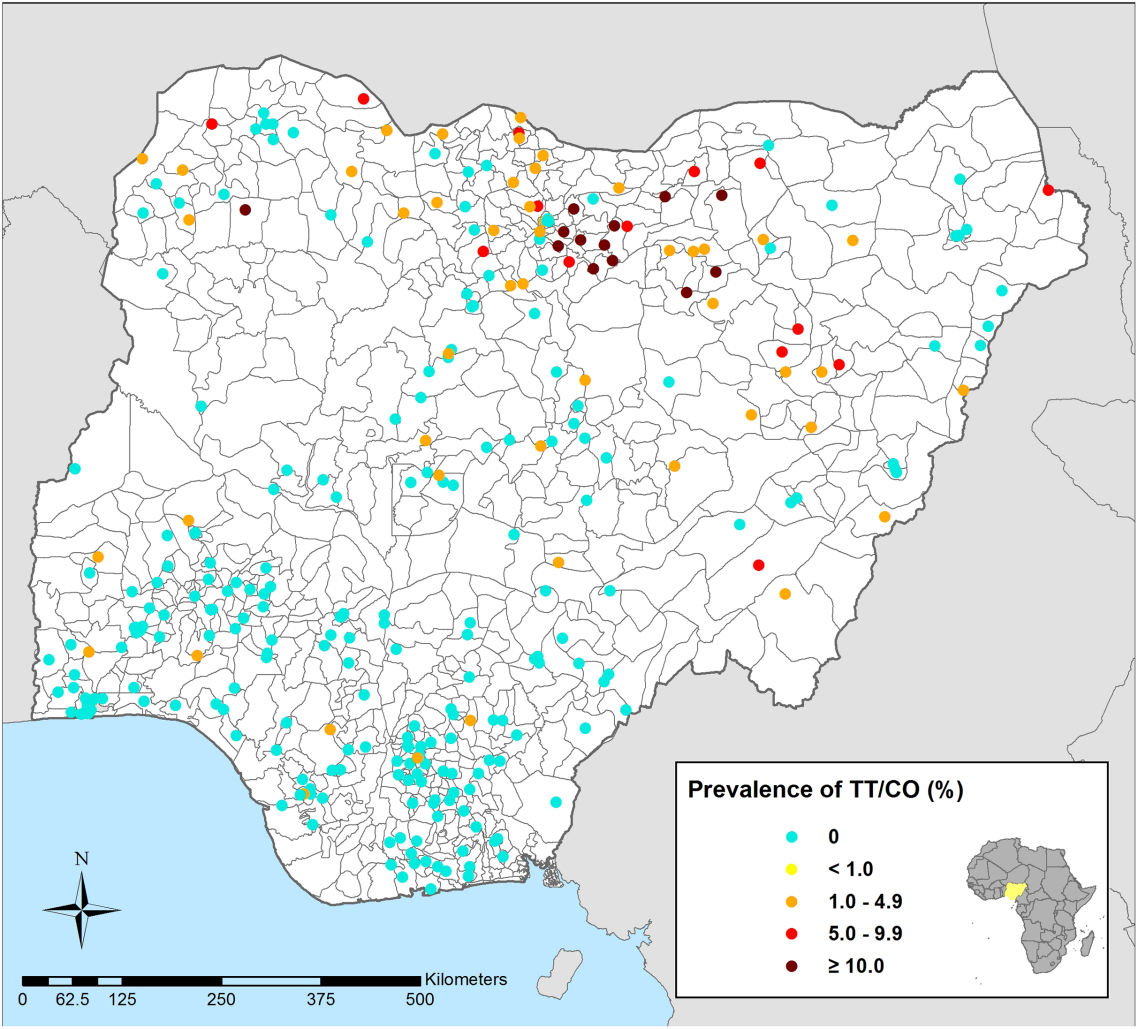
Prevalence of trichiasis (TT) or corneal opacity (CO) in adults over 40 years in Nigeria, 2005–2007. Higher prevalence clusters are predominantly in northern areas of Nigeria.

Complete geolocated survey data were available for 304 clusters, from which 13,543 individuals aged 40 years and above, resident in 8,621 households, were examined for TT and CO. Overall, 198 (adjusted prevalence: 1.45%) individuals were diagnosed with either TT or CO in at least one eye, and only two individuals had clinical signs of CO without concurrent TT. Fig 1 presents the distribution of TT/CO among adults aged 40 years and above within clusters (prevalence ranging from 0 to 28.9%) and highlights the greater burden of trachoma in the northern areas of Nigeria.

Summary characteristics of the study population are described in Table 2 and reflect socioeconomic trends across the country. Overall, only 10% of participants used a flush toilet although 64% had access to a pit latrine, and over half (56%) of the participants could not read. Areas within northern geopolitical zones had higher illiteracy (62.5%) and unemployment (19.6%) compared to southern zones (49.3% and 12.6%) (adjusted p-values <0.0001). Although fewer people had access to a flush toilet in the northern zones (4.4%) than southern zones (17.5%) (adjusted p-value <0.0001), open defecation was also reported less in northern zones (20.9%) compared to the south (31.3%) (adjusted p-value 0.008).

**Table 2:** Descriptive statistics of the 13,543 individuals included in the 2007 National Blindness and Visual Impairment Survey in Nigeria, 2005–2007.

Statistic	Number (%)
Total number individuals	13,543
Gender	
Female	7,317 (54.0)
Age group:	
40–50 years	5,821 (43.0)
50–60 years	3,371 (24.9)
60–70 years	2,600 (19.2)
70–80 years	1,312 (9.7)
>80 years	439 (3.2)
Literacy:	
Easily	2,925 (21.6)
With difficulty	2,983 (22.0)
Not at all	7,635 (56.4)
Occupation:	
Professional	1,317 (9.7)
Semi Skilled	10,013 (73.9)
Unemployed	2,213 (16.3)
Latrine type:	
Flush	1,415 (10.4)
Pit Latrine	8,648 (63.9)
Bush	3,480 (25.7)
Improved water source:	
Unimproved	3,802 (28.1)
Improved	9,741 (71.9)
Proximate water source:	
Wells/boreholes/surface water	8,188 (60.5)
Within street or household	5,355 (39.5)
TT and/or CO	198 (1.5)

TT: trachomatous trichiasis; CO: trachomatous corneal opacity

### Exploratory analysis

**Fig 2:**
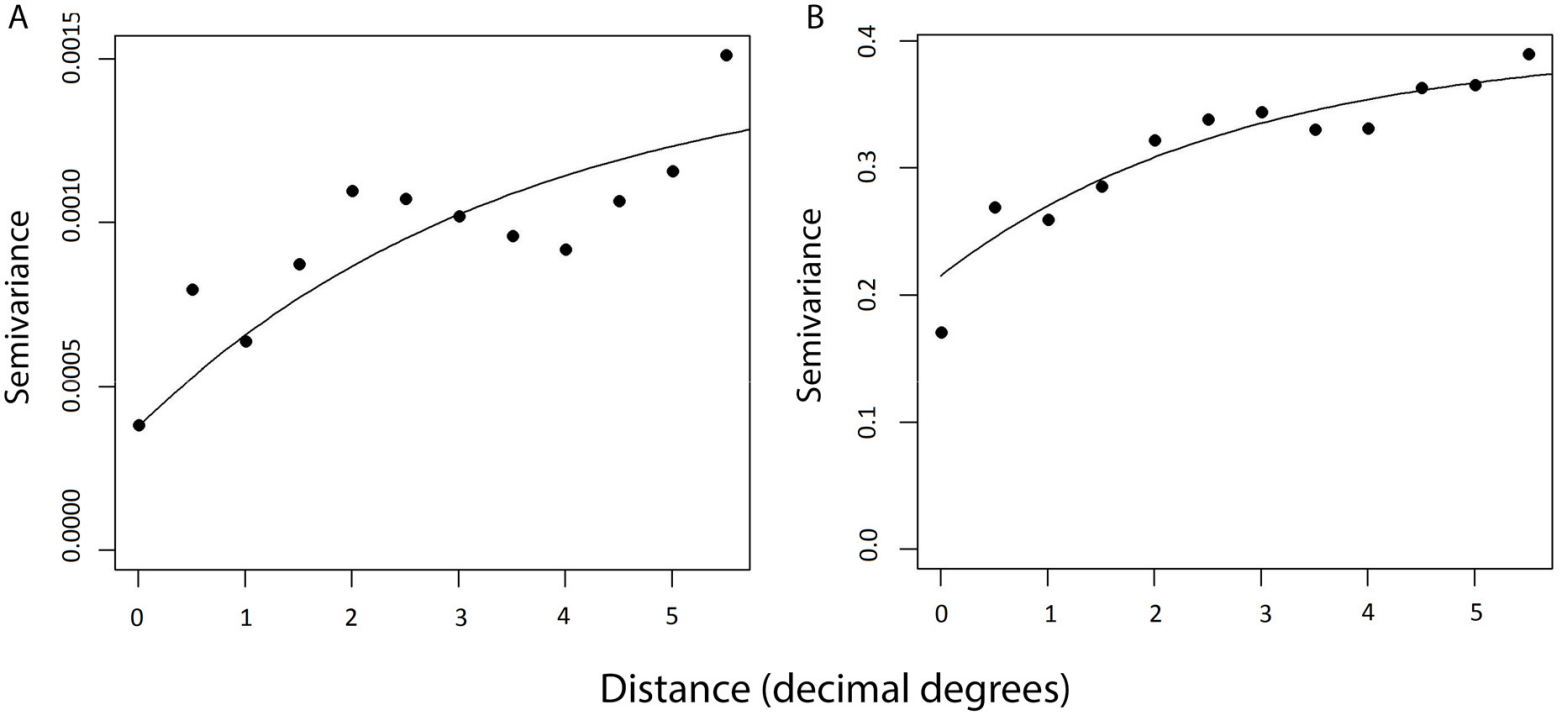
Semi-variograms related to risk mapping models for presence of trachomatous trichiasis (TT) or corneal opacity (CO) in adults over 40 years, Nigeria, 2005–2007. There is evidence of spatial structure and the suggestion of large scale trends in risk of TT/CO both in the raw data (A) and graphs of Pearson’s residuals from model 2 including individual and cluster-level covariates (B).

All field collected variables and environmental covariates were strongly associated with the presence of TT/CO in univariate logistic regression models, with the exception of mean annual temperature, as summarised in Table 3. Correlation was observed between a number of variables related to socioeconomic status, including occupation, water availability, literacy and latrine type. Literacy was associated both with occupation (p<0.0001) and gender (p<0.0001). Women with a lower literacy status had a higher risk of TT/CO, partly accounting for the increased risk observed in illiterate individuals. A geographic north-south trend in risk of trichiasis was apparent across the country, and the unbounded semi-variogram for the raw TT/CO prevalence supported the presence of spatial autocorrelation in the distribution of disease (Fig 2A).

**Table 3:** Univariate associations with variables in 304 clusters of 13,543 individuals aged ≥ 40 years in Nigeria, 2005–2007.

Variables		Group	OR (p-value)
***Field-collected variables***	**Individual level**		
	Age group:	40–49 years	1
		50–59 years	1.68 (0.02)
		60–69 years	4.00 (<0.0001)
		70–70 years	4.65 (<0.0001)
		80+ years	5.30 (<0.0001)
	Gender	Female	2.61 (<0.0001)
	Literacy	Easily	1
		Difficult	4.11 (<0.0001)
		Not at all	2.06 (0.03)
	Improved water source	Unimproved	1
		Improved	1.40 (0.421)
	Proximate water source	Village or further	1
		Within street or household	0.95 (0.86)
	Occupation	Professional	1
		Semi Skilled	11.44 (0.03)
		Unemployed	35.55 (0.002)
	**Cluster level**		
***Climatic covariates***	Mean annual precipitation (mm)		0.997 (<0.0001)
	Monthly Average land surface temperature (LST)		1.358 (<0.0001)
	Mean annual temperature (°C)		1.01 (0.59)
***Environmental covariates***	Altitude (meters)	<200	1
		200–499	14.41 (<0.0001)
		500 +	5.64 (<0.0001)
	Enhanced vegetation index (EVI)	≥ 0.35	1
		0.25–0.34	6.66 (0.001)
		0.15–0.24	26.00 (<0.0001)
		< 0.15	4.53 (0.01)
	Land cover type	Other	1
		Savannah/Grasslands	2.70 (<0.0001)
	Urban classification	Rural	1
		Urban	0.27 (<0.0001)
	Distance to surface water (km)		1.18 (<0.0001)
	Population density (per 5km cell)		0.99 (<0.0001)
	Ruminant density (per 5km cell)		1.003 (0.02)

All associations adjusted for clustering within villages

The results from the PCA identified five key groupings of variability in climatic covariates, from each of which a single variable was retained. Mean annual precipitation and land surface temperature were retained from the two contrasting groups from the first component. Mean annual temperature and altitude (identified in the PCA as a second collinear pair contributing to climatic variation) and EVI were also retained for further analyses with all other uncorrelated environmental indices. Summary statistics for these variables are presented in Table 1. During model building, the residual variation in EVI was initially significant after accounting for collinear effects of precipitation and LST, but dropped out after accounting for urban classification.

### Geostatistical risk model

Bayesian hierarchical models retained both individual and cluster-level covariates (Table 4). The final model reported is Model 2, which is non-spatial and includes age, gender, and occupation as well as mean annual precipitation, residual variation in LST, mean annual temperature and urban classification. Risk of TT/CO increased with age and was higher in women than men (OR 2.46, 95% BCI 1.82–3.39). Lower socioeconomic status, as measured by occupation, was also associated with an increased risk of trichiasis. Despite wide confidence intervals, there was evidence that individuals employed in a professional capacity had the lowest risk of trichiasis while the unemployed were at highest risk (OR 16.71, 95% BCI 3.23–556.1).

**Table 4:** Random-effects models for trachomatous trichiasis or corneal opacity in adults over 40 years in Nigeria.

Variable	Null Model 1^a^ OR (95% BCI)	Null Model 2^b^ OR (95% BCI)	Model 1^a^ OR (95% BCI)	Model 2 ^a^ OR (95% BCI)	Model 3^b^ OR (95% BCI)
Age:					
40–49 years			-	-	-
50–59 years			1.78 (1.27, 2.52)*	1.86 (1.33, 2.71)*	1.90 (1.30, 2.81)*
60–69 years			3.99 (2.87, 5.85)*	4.48 (3.18,6.34)*	4.54 (3.17, 6.55)*
70–79 years			4.30 (2.68, 6.61)*	4.78 (3.13, 7.43)*	5.17 (3.25, 8.05)*
80 plus years			3.70 (1.76,7.36)*	5.24 (2.51, 10.67)*	5.62 (2.63, 10.98)*
Gender:					
Male			-	-	-
Female			2.23 (1.61, 3.01)*	2.46 (1.82, 3.39)*	2.55 (1.89, 3.51)*
Occupation:					
professionals			-	-	-
semi/skilled			9.80 (2.09, 161.27)*	10.83 (2.18, 359.4)*	15.39 (3.18, 116.1)*
unemployed			17.84 (3.89, 286.20)*	16.71(3.23, 556.1)*	21.63 (4.34, 173.2)*
Mean annual precipitation				0.17 (0.10, 0.26)*	0.21 (0.11, 0.38)*
Residual LST				2.95 (1.36, 6.85)*	1.91 (0.79, 5.08)
Squared term				0.21 (0.07,0.58)*	0.19 (0.05, 0.63)*
Mean annual temperature				0.89 (0.69, 1.14)	0.91 (0.62, 1.37)
Squared term				0.75 (0.62, 0.87)*	0.83 (0.67, 1.02)
Urban classification				0.27 (0.13, 0.52)*	0.34 (0.17, 0.61)*
	Beta coefficient (95% BCI)	Beta coefficient (95% BCI)	Beta coefficient (95% BCI)	Beta coefficient (95% BCI)	Beta coefficient (95% BCI)
Intercept	-5.86 (-6.47, -5.36)	-5.43 (-6.58, -4.35)	-9.71(-12.54, -7.97)	-9.19 (-12.55, -7.47)	-9.90 (-12.1, -8.29)
σ^2^	4.04 (2.56, 6.20)	0.52 (0.23, 1.04)	4.66 (3.35, 6.55)	1.53 (0.98, 2.41)	0.006 (0.00, 0.41)
Spatial southern ϕ [range in km]		0.22 (0.05, 0.55) [1527]			59.13 (22.2, 94.19) [6]
Spatial southern σ^2^		5.72 (1.13, 19.41)			1.27 (0.24, 4.18)
Spatial northern ϕ [range in km]					0.92 (0.23, 2.42) [365]
Spatial northern σ^2^					2.55 (1.18, 8.00)
DIC	1658	1610	1533	1490	1483

OR: odds ratio; BCI: Bayesian credible interval; DIC: deviance information criterion (smaller DICs indicate better model fit); ϕ = rate of decay of spatial correlation; σ^2^ = variance of random effect

^a^ Non-spatial random effect only;

^b^ Including separate spatial random effect for northern areas of Nigeria (North-East and North-West zones)

Increased precipitation was associated with a lower risk of TT/CO in Nigeria (OR 0.17, 95% BCI 0.06–0.33), and higher residual LST was uniquely associated with an increased risk of TT/CO (OR 2.95 95% BCI 1.36–6.85) additional to the contribution it made through its relationship with precipitation. Although not identified as a risk factor in the univariate analyses, increased mean annual air temperature was associated with lower risk of TT/CO after controlling for the effects of other environmental factors. This variable was kept in the model based on the lower DIC. Finally, the odds of TT/CO were lower in urban areas (OR 0.27 95% BCI 0.13–0.52), after controlling for individual-level risk factors. Approximation of the VPC using a simulation approach suggested that 14% of the total variation (based on a null model) was attributed to the cluster level. After inclusion of both individual and cluster-level risk, only 0.7% of the overall residual variation was at the higher level.

**Fig 3:**
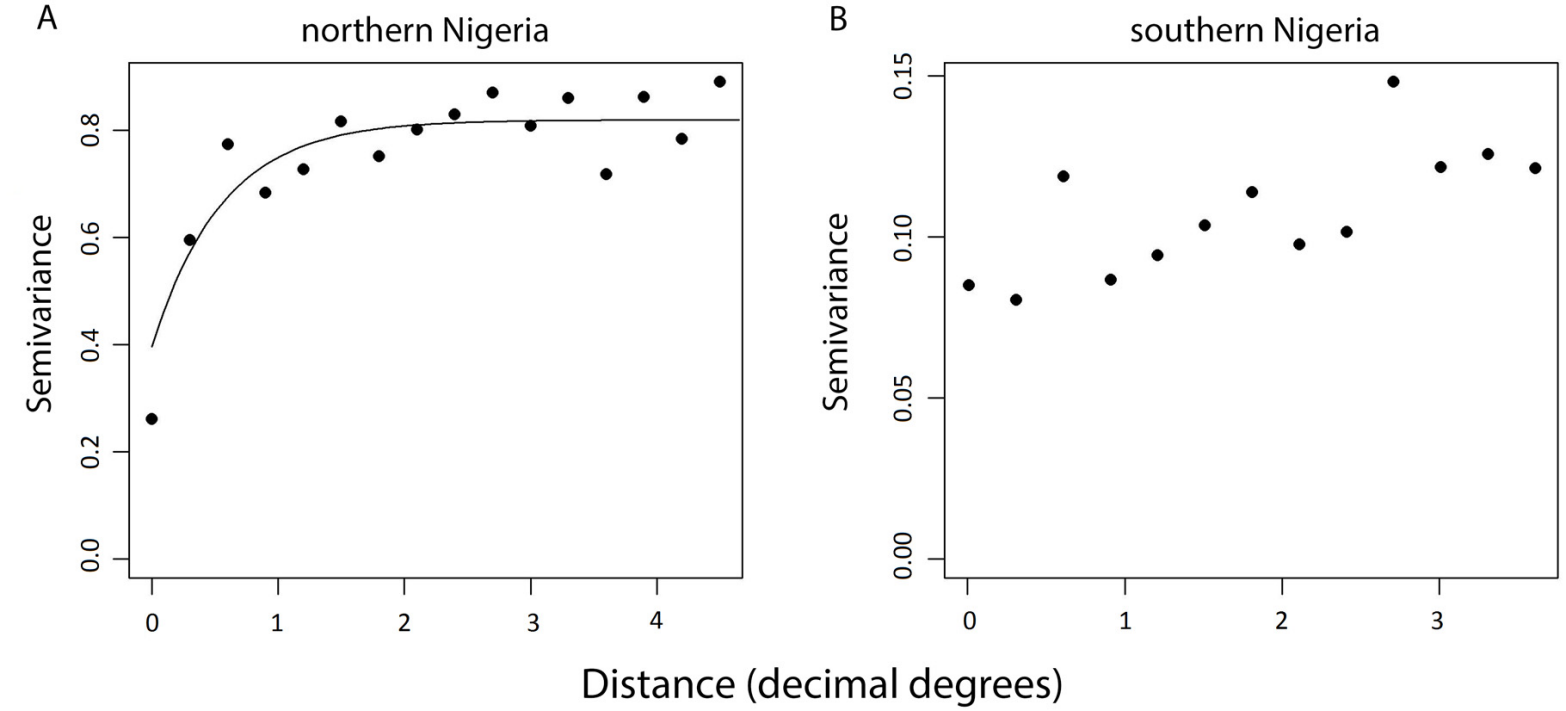
Semi-variograms of the spatial structure of trachomatous trichiasis (TT) or corneal opacity (CO) present in residuals from nonspatial Model 2 in northern (A) and southern (B) regions of Nigeria. While there is no evidence of spatial structure in the residuals from southern Nigeria, the semi-variance (or difference) in risk is observed to increase with distance in northern Nigeria. Of key interest is the ratio of sill to nugget variance, which provides information on how spatially structured the variance in prevalence is. In northern Nigeria the ratio is 2.07, suggesting that just over half of residual variance is spatially structured. This structure may be due to dependency on unknown risk factors which are locally clustered in these areas or non-stationarity in the relationships between observed risk factors and disease.

**Fig 4:**
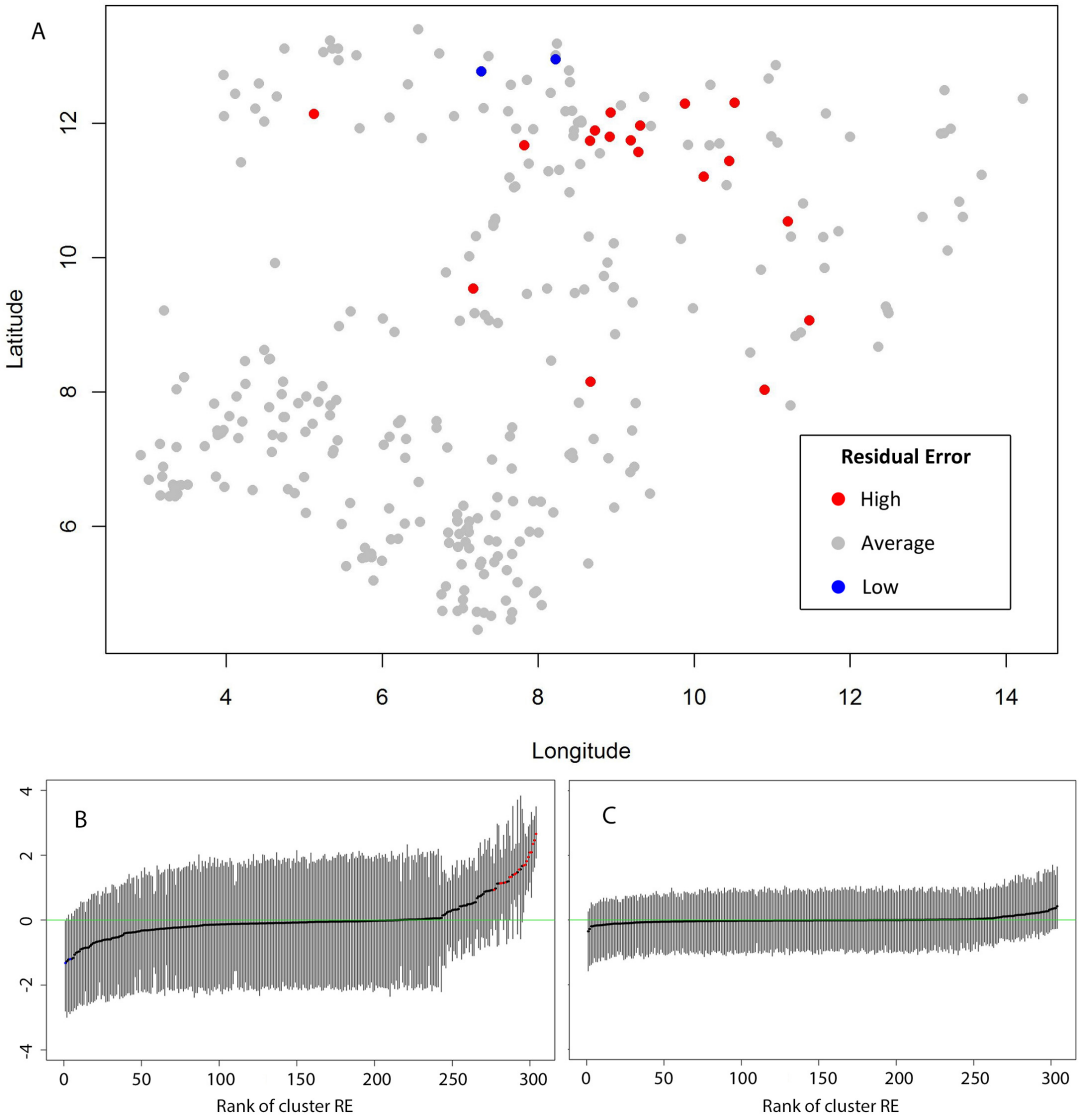
Mapped residuals from non-spatial Model 2 with 95% Bayesian credible intervals (BCI). Lines that are above zero indicate that areas of high residual risk are localised in northern Nigeria, with a large cluster present encompassing southern Jigawa, eastern Kano and northern Bauchi states (A and B). In contrast, residuals from spatial Model 3 (C) have narrower confidence intervals, which include zero.

Although the results from the non-spatial model are reported here, there was evidence of large scale spatial trends as well as local clustering of TT/CO risk in Nigeria. The semi-variogram of the Pearson’s residuals from Model 1 indicated that, compared to the null model, the addition of covariates decreased the proportion of variation that was spatially structured and controlled for large-scale trends (Fig 2). This residual spatial structure varied within Nigeria (non-stationarity), with a higher proportion of residual variation in North-East and North-West zones showing spatial structure (Fig 3). Graphs and maps of the residuals from the non-spatial Model 2 suggested that residual variation was localised in a large cluster of higher risk in the north of Nigeria (Fig 4A and 4B). Inclusion of a separate random effect for these northern zones had the effect of reducing overall residual error in the model, as indicated by the reduction in the variance of the non-spatial random effect and narrower confidence intervals (Model 3, Table 4 and Fig 4C). However, addition of these terms also reduced observed associations with LST and mean annual air temperature, and widened their confidence intervals. This finding suggests that while these environmental factors may be associated with the distribution of risk in the north, they do not explain all observed clustering and are made redundant by inclusion of a spatial random effect. The range of spatial autocorrelation can be calculated by 3/ϕ and is thus 3.26 decimal degrees (approximately 365 km) in the north. Residual variation in the south was more likely to be aspatial and due to individual level factors.

## Discussion

The present study provides evidence that both individual-level risk factors and broader climatic conditions are associated with later stages of trachoma in adults over the age of 40 years in Nigeria, using uniquely detailed national survey data. The hierarchical approach used in this analysis has the advantage of incorporating risk factors at multiple levels and explicitly modelling residual spatial correlation in TT/CO that could affect the standard errors of estimates of association. A number of well-established individual-level risk factors for trichiasis were identified that included age, gender and occupation, as well as large-scale climatic and environmental factors (precipitation, LST, temperature and urban classification) that explained further variation in risk across the country. After adjusting for these factors, there remained a large cluster of higher risk localised in northern Nigeria (North-East and North-West zones). This finding suggests the presence of unknown risk factors which are locally clustered in these areas or spatially-varying relationships between included covariates and disease.

Individual-level factors found to be associated with trichiasis are consistent with our general understanding of trachoma epidemiology. These associations replicate those previously found in a number of studies in Nigeria [55], other countries in sub-Saharan Africa [23,28,56] and trachoma endemic areas worldwide [57–59]. The risk of TT increases with age, presumably due to cumulative scarring caused by repeated infection over an individual’s life, while the higher risk in females is commonly attributed to close contact with children and greater exposure to infection with the causative agent [3,60]. Occupation is a characteristic that captures various dimensions of an individual’s socioeconomic status (SES) and may be linked to underlying risk factors for infection, including hygienic behaviours, use of water, human waste disposal, overcrowding or other conditions that encourage the proliferation of flies or increased transmission through contact and fomites. Previous studies have also shown a greater risk of trachoma to associate with lower scores on various socioeconomic measures, including occupation [61], literacy or formal schooling [59,62]; or measures of living standards; but not uniformly across all settings [63]. Variation in relevant socioeconomic measures between settings may reflect differences in underlying transmission dynamics, equity in access to treatment and surgical interventions, as well as unreliability of the measures themselves. After accounting for these risk factors, living in urban areas remained associated with a lower risk of trichiasis. This finding supports anecdotal evidence underlying current trachoma survey strategies that exclude urban areas, and may reflect reduced access to health services or increased contact with flies associated with rural lifestyles.

After controlling for individual-level risk factors, lower precipitation, higher land surface temperatures and lower mean annual temperatures were associated with a higher risk of TT in Nigeria. On this scale, climatic factors may influence transmission dynamics through hygienic behaviours related to perceived water availability, actual water availability or as determinants of fly abundance, biological fitness and behaviour [13,26,29,30,64,65]. Shared variation in precipitation and LST accounted for the most climatic variation, and might be interpreted as variation common to different measures of climatic water availability. Higher LST, after accounting for collinear variation with precipitation, was associated with a further increased risk of TT/CO. These findings are consistent with previous analyses associating a higher risk of active trachoma with higher aridity and lower rainfall [20,21,23,66,67]. The higher risk of TT/CO associated with lower air temperatures (or higher altitudes) seems counter-intuitive, however this association has been reported in previous studies with limited control for potentially confounding variables [22,28]. Lower temperatures are hypothesized to have a biological effect on the fly vector, *M sorbens*, the life span of which ranges from 12 days at 32°C to 35 days at 24°C [64]. Toyama et al. (1981) also suggested a mechanism by which increased land surface temperature might be associated with a greater fly abundance [17]. This study recorded temperatures beneath dung pats, and associated higher temperatures with formation of crusts that are hypothesised to protect larvae from predation. It is expected that TT/CO in adults who were ≥40 years old at the time of the survey mainly reflects exposure to risk factors influencing transmission decades previously (assuming little population movement across clusters/climatic gradients). However, it is possible that certain climatic factors may also influence the development or subsequent evolution of trachomatous scarring and hence TT/CO. Ongoing active disease and eye irritants like ocular dryness may be associated with drier climatic conditions and contribute to chronic conjunctival inflammation. This in turn has been associated with a higher risk of TT and faster progression to later disease stages [2,18–20].

Despite strong links between water availability and transmission of trachoma, there are a number of potential reasons why water source was not identified as a risk factor in this analysis. First, domestic water consumption and, importantly, its allocation for hygienic purposes will mediate any relationship between water availability and trachoma [30]. Water allocation is difficult to measure, and while distance to water [26,68,69] and type of source [70] have been associated with trachoma in some studies, they are at best proxy measures of household and individual water use. It is likely that our classifications of water source were not able to capture relevant measures. In addition, a study on water use patterns in Tanzania highlighted the importance of perceived water availability and its impact on water usage, rather than availability itself [29]. It is possible that perceptions around water availability are partly driven by climatic experiences and thus may influence subsequent behaviours, including allocation within the household. Second, this survey was done over 30 months and limited evidence suggests that the water source reported as “main” may vary seasonally in Nigeria [71]. Consequently, any observed relationship between distance and usage may be stronger in the dry season. Third, individual occupation as a socioeconomic measure may have captured any effect of water source, as those with higher incomes had improved water access. And finally, while water is likely to be associated with transmission, trichiasis prevalence is likely to more strongly reflect historical transmission levels, prior to any recent interventions or secular trends. In support of this hypothesis was our finding that an improved water source was associated with an increase in the unadjusted odds of disease in the driest areas, potentially reflecting targeting of water interventions to the driest areas in the last 20–30 years.

One of the strengths of this analysis lies in its explicit recognition of the hierarchical structure of the data and ability to incorporate residual spatial variation. After accounting for risk factors at the individual and cluster level, there was evidence that TT/CO was spatially structured over a large (365 km) range in the north. This is likely to be due to a large cluster of residual risk, focused around southern Jigawa, eastern Kano and northern Bauchi states. Approximation of the VPC using a simulation approach suggested that 14% of the total variation was attributed to the cluster level. After accounting for risk factors at both levels, this was reduced to less than 1%. This suggests that risk of TT/CO is more variable within clusters than between clusters, and is consistent with the natural history of trachoma which requires repeated infections of *C trachomatis*, observed to cluster within households [6,28] and individuals [72]. In contrast, a recent study by Hagi et al. attributed nearly 40% of observed variation in active trachoma to the village level [9]. It is not clear what approach was used for this estimate, thus it may not be directly comparable to estimates from this study, but a higher proportion of variation between villages may reflect the importance of environmental factors on transmission dynamics via flies, and water availability. The influence of these factors may give a relatively homogenous “spread” of active disease risk across a community, whereas clustering of TT may reflect the importance of individual-level risk factors which influence the predisposition to infection, duration of infection, or immunological response to infection over longer periods of time.

Despite the robust approach used to model these data, there are a number of limitations inherent in the data and methods. First, as anticipated, strong collinearity in environmental, climatic and socioeconomic variables across the country placed limitations on our ability to disentangle their independent effects. Observed associations with climatic factors may reflect socioeconomic factors that we have not taken into account, as rural populations are likely to be dependent on agro-ecological conditions for crop and livestock productivity. Second, this survey was cross-sectional and TT is a condition that represents the cumulative effect of many infections over time. Thus potential decadal climate variability, migration during an individual’s adult life or variation in other risk factors for TF between the period of exposure and time of survey limits any inferences of causality. Expected associations may be masked, or even reversed in some cases, where access to the SAFE strategy (including surgery and environmental improvement) has been implemented in high transmission areas. Third, the sample used for this analysis was developed for a national cross-sectional blindness and low vision prevalence survey, and thus may not be an ideal sample to extensively study risk factors for a low prevalence condition like trichaisis. And finally, not all points were able to be geolocated to a specific point location (7%) and the distribution of these points varied across the country. For example, a high proportion (24%) of points were geolocated to the LGA centroid in the North East zone and a low proportion (0%) in the North Central zone. Errors in geolocation could introduce misclassification of cluster-level environmental data and biases in the analysis and interpretation of results. However, exclusion of points geolocated to the centre of LGAs had a minimal effect on associations in the model.

For the first time, we have quantified associations between climatic factors and risk of TT/CO in Nigeria while accounting for the effects of individual-level risk factors and residual spatial dependency. While the results indicate that individual-level factors are an important source of variation, individuals living in drier and rural areas of Nigeria were at greater risk of chronic disease stages. This supports anecdotal evidence associating limited water availability with trachoma although other mechanisms may also be important, such as the effect of temperature on the abundance, breeding potential and activity of *M sorbens* [73]. The current focus on WASH interventions to combat other neglected tropical diseases (NTDs), such as soil-transmitted helminths and schistosomiasis, provides opportunities to strengthen water availability and its use for hygienic purposes, which will have an added impact on the burden of trachoma. Findings from this study may help to more reliably extrapolate trichiasis data within countries and regions and refine estimates of the burden of disease, although further work is required to investigate associations at larger scales and in different endemic contexts. A better understanding of the distribution of the burden of trichiasis and underlying risk factors in Nigeria may aid scale-up of outreach and targeting of surgical interventions.

## Supporting Information

S1 Table Description of climatic and environmental variables. Description and source of climatic and environmental data used in the analyses.
(DOCX)

S1 Fig Maps of climatic and environmental factors within in Nigeria, as detailed in S1 Table. (DOCX)
