# Supplementary material for: Multilevel Analysis of Trachomatous Trichiasis and Corneal Opacity in Nigeria: The Role of Environmental and Climatic Risk Factors on the Distribution of Disease
Source: PLoS Negl Trop Dis. 2015 Jul 29;9(7):e0003826. doi: 10.1371/journal.pntd.0003826 (PMC4519340; doi:10.1371/journal.pntd.0003826)
Supplement: S1 Table — Description and source of climatic and environmental data used in the analyses. (DOCX) [file pntd.0003826.s001.docx]

|  | |
| --- | --- |
| **Variable** | **Description and source** |
| **Climate** |  |
| Mean Annual Temperature (°C) | Interpolation of average monthly mean annual temperature data (~1950-2000)  Derived: Meteorological stations  Resolution: 2.5 arc-minute (~5 km)  Source: WorldClim BioClim variables |
| Max Warmest Temperature (°C) | Interpolation of average maximum temperature in the warmest month (~1950-2000)  Derived: Meteorological stations  Resolution: 2.5 arc-minute (~5 km)  Source: WorldClim BioClim variables |
| Mean Annual Precipitation (mm) | Interpolation of average monthly mean annual precipitation data (~1950-2000)  Derived: Meteorological stations  Resolution: 2.5 arc-minute (~5 km)  Source: WorldClim BioClim variables |
| Precipitation of Driest Month (mm) | Interpolation of average monthly mean annual precipitation data (~1950-2000)  Derived: Meteorological stations  Resolution: 2.5 arc-minute (~5 km)  Source: WorldClim BioClim variables |
| Annual aridity index | Interpolation of mean Annual Precipitations/Mean Annual Potential Evapo-Transpiration (~1950-2000).  Derived: Meteorological stations (WorldClim bioclimatic variables)  Resolution: 30 arc second (~1 km)  Source: Consortium for Spatial Information (CGIAR-CSI)  Global-Aridity and Global-PET Database [71,72] |
| Monthly average Potential Evapo-Transpiration (PET) (mm/month) | Interpolation of monthly average measure of the amount of evaporation that would occur if a sufficient water source were available based on the Hargreaves model using monthly average geo-datasets of: mean temperature, daily temperature range and extra-terrestrial radiation (~1950-2000)  Derived: Meteorological stations (WorldClim bioclimatic variables)  Resolution: 30 arc second (~1 km)  Source: Consortium for Spatial Information (CGIAR-CSI)  Global-Aridity and Global-PET Database [71,72] |
| **Environmental** |  |
| LST (°C) | Mean annual land surface temperature for the years 2005-2007.  Derived: Satellite remote sensing  Resolution: 2.5 arc-minute (~5 km)  Source: Moderate Resolution Imaging Spectroradiometer (MODIS) on NASA’s Terra satellite |
| Altitude (meters) | Elevation data  Derived: Radar  Resolution: 2.5 arc-minute (~5 km)  Source:  Shuttle Radar Topography Mission (SRTM) |
| Enhanced Vegetation Index (EVI) | Fourier-transformed index of the vegetation signal from surface reflectances  Derived: Satellite remote sensing  Resolution: 2.5 arc-minute (~5 km)  Source: Moderate Resolution Imaging Spectroradiometer (MODIS) on NASA’s Terra satellite |
| Global land cover classification^a^ | Global land cover classification  Derived: Satellite remote sensing  Resolution: 2.5 arc-minute (~5 km)  Source: UN *Land Cover Classification* System (LCCS) using ENVISAT satellite mission's MERIS sensor at 5km^2^ resolution |
| Ruminant density (animals per 5km cell) | Predicted distribution of livestock in 2005.  Derived: Observed livestock statistics and environmental variables  Resolution: 3 arc-minute (~5 km)  Source: FAO Global Livestock Densities [73] |
| Cost-Distance to road network | Accessibility measure calculated as a cost-distance surface using a set of topographical variables set as constraints to access of the road network.  Derived: Distance to road network, slope, major water bodies, streams and land cover  Resolution: 30 arc second (~1 km)  Source: Generated for this analysis |
| Distance to river or water body | Derived: Distance to nearest river or surface water body  Resolution: 2.5 arc-minute (~5 km)  Source: FAO Rivers and Surface Water Bodies database |
| Urban classification | Gridded database of urban settlements with populations greater than 1000 persons.  Derived: Satellite night-light data and gridded population data  Resolution: 30 arc second (~1 km)  Source: Global Rural-Urban Mapping Project (GRUMP) |
| Population density | Gridded population data for the year 2010  Derived: NA  Resolution: 30 arc second (~1 km)  Source: SEDAC’s Gridded Population of the World, Version 3 data set (GPWv3) |
| ^a^Classified as binary variable indicating savannah/grasslands | |
