# Supplementary figures and images for: Multilevel Analysis of Trachomatous Trichiasis and Corneal Opacity in Nigeria: The Role of Environmental and Climatic Risk Factors on the Distribution of Disease
Source: PLoS Negl Trop Dis. 2015 Jul 29;9(7):e0003826. doi: 10.1371/journal.pntd.0003826 (PMC4519340; doi:10.1371/journal.pntd.0003826)

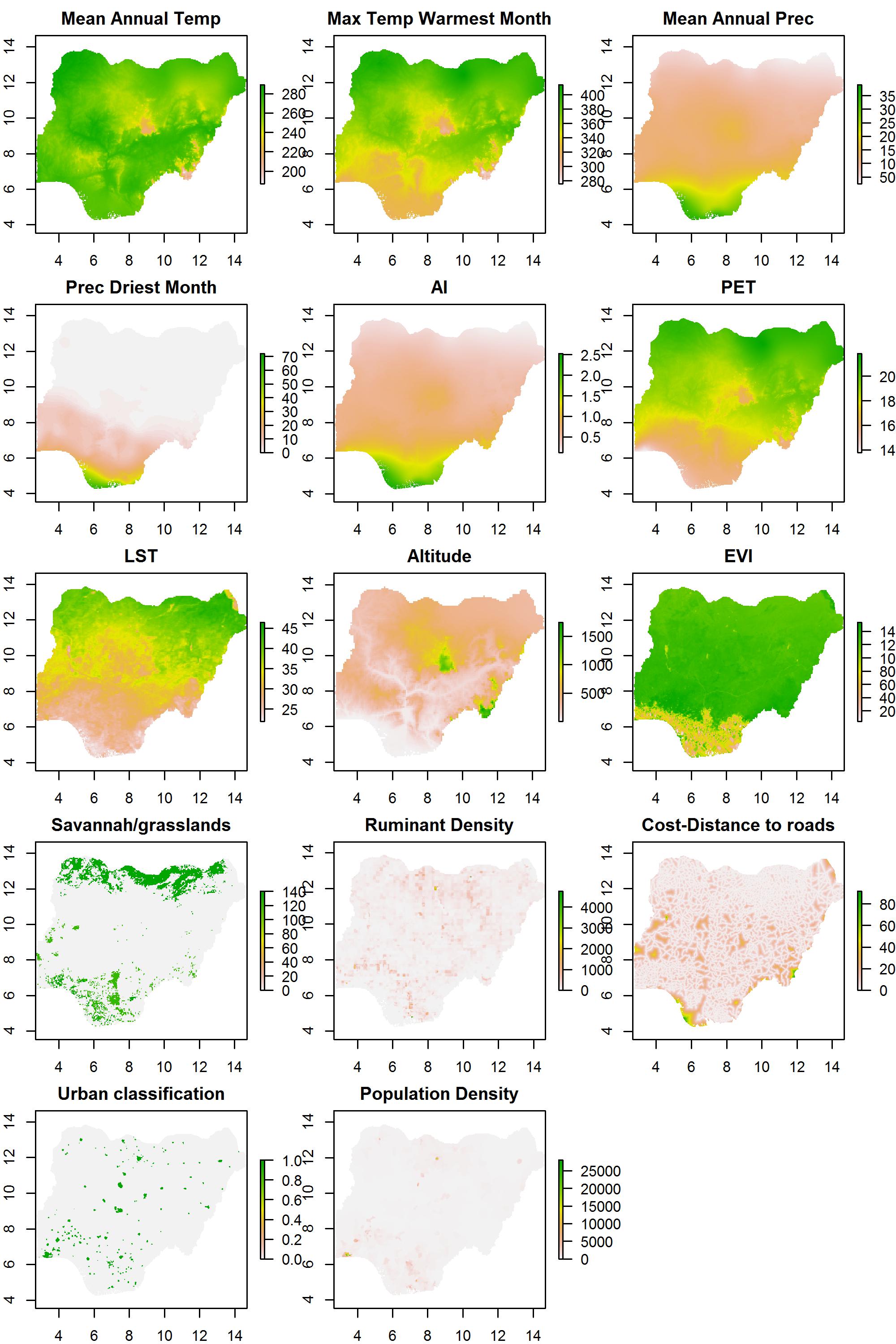

Supplement: S1 Fig — (DOCX) [file pntd.0003826.s002.docx]
